# Joint Involvement Can Predict Chikungunya in a Dengue Syndemic Setting in India

**DOI:** 10.1007/s44197-023-00163-8

**Published:** 2023-11-14

**Authors:** Abhinav Sinha, Deepali Savargaonkar, Auley De, Aparna Tiwari, C. P. Yadav, Anupkumar R. Anvikar

**Affiliations:** ICMR-National Institute of Malaria Research, New Delhi, India

**Keywords:** Joint pain, Joint swelling, Chikungunya, Dengue, India

## Abstract

Dengue and chikungunya have been endemic in India but have the tendency to cause periodic epidemics, often together, wherein they are termed ‘syndemic’. Such a syndemic was observed in 2016 in India which resulted in a further scarcity of already resource-poor specific diagnostic infrastructure even in many urban conglomerates. A cross-sectional study was thus conducted, on 978 fever patients that consulted the ICMR-NIMR fever clinic, New Delhi, in September 2016, with an objective to identify symptom/s that could predict chikungunya with certainty. The overall aim was to rationally channelize the most clinically suitable patients for the required specific diagnosis of chikungunya. Based on their clinical profile, febrile patients attending NIMR’s clinic, appropriate laboratory tests and their association analyses were performed. Bivariate analysis on 34 clinical parameters revealed that joint pain, joint swelling, rashes, red spots, weakness, itching, loss of taste, red eyes, and bleeding gums were found to be statistically significantly associated predictors of chikungunya as compared to dengue. While, in multivariate analysis, only four symptoms (joint pain in elbows, joint swelling, itching and bleeding gums) were found in statistically significant association with chikungunya. Hence, based on the results, a clinician may preferably channelize febrile patients with one or more of these four symptoms for chikungunya-specific diagnosis and divert the rest for dengue lab diagnosis in a dengue–chikungunya syndemic setting.

## Background

Arboviral diseases (AVDs) contribute to sizeable burden globally [1] and particularly dengue and chikungunya have major public health importance in India. With similar agent-host-vector-time relationships, it is highly plausible that dengue and chikungunya viruses co-circulate in the same geo-temporal dimension and generate a febrile syndemic leading to clinical ambiguity in specific diagnosis [2, 3]. General clinical features of dengue and chikungunya are so indifferent that these diseases are often collectively named as dengue/break bone fever or chikungunya fever is even misdiagnosed as dengue fever [4, 5] further aggravating the diagnostic dilemma [6].

Chikungunya virus (CHIKV) usually manifests clinically as an acute self-remitting triad of fever, pruriginous maculopapular rash, and arthralgia (joint pains). Although arthralgia has been noted as a clinical ‘marker’ of chikungunya [7, 8], reports have debated this due to variability of its presentation in chikungunya [9], its presence in other AVDs [10], including dengue [11], and also due to variations in clinical acumen in diagnosing arthralgia vis-à-vis arthritis, rheumatism, and musculoskeletal pains. The chikungunya-associated arthralgia generally is a migratory polyarthralgia with pain on movement, involves small/large joints of extremities, and is usually symmetric [12–16]. Persistent arthralgia (≥ 3 months) has some differential diagnostic importance but its clinical applicability is limited due to recall bias and association with non-infectious co-morbidities [17, 18]. Confirmatory lab diagnosis is important as non-steroidal anti-inflammatory drugs are contraindicated in dengue, but can be safely prescribed for chikungunya-associated arthralgias.

Routine laboratory diagnoses are non-specific. Enzyme-linked immunosorbent assays (ELISA) for IgM and detection of viral gene/s (E1 by RT-PCR) are helpful but their interpretations warrant thorough understanding of period of viremia and immunity [19, 20] as DENV NS1 is detectable up to 5 days and DENV/CHIKV IgM is detected only after 5–7 days of symptom onset and there are concerns with cross-reactivity between closely-related viruses. Additionally, IgM antibodies remain detectable for up to 3 months after symptoms develop [21, 22]. CHIKV RNA by RT-PCR [23–26] is positive only within 3–6 days of onset [27] and its use is limited as most patients report late to health facilities. Virus isolation [28] remains the gold-standard confirmatory diagnosis and PCR-based tests are costly and have high diagnostic turnover time. A few rapid point-of-care diagnostics are available but their utility is limited by poor performance [29, 30].

The problem is magnified many-folds in resource-poor settings as they bear the major brunt of AVDs. Developing a clinical diagnostic algorithm can increase the specificity of a likely diagnosis [31] and channelize selected patients for confirmatory diagnosis that aid in efficient allocation of resources. Therefore, the aim of this study was to identify certain clinical features that could predict chikungunya over dengue in a syndemic setting.

## Methods

Approval (NIMRWEC/2021/06–01) to perform analysis was taken from the Institutional Ethics Committee of ICMR-National Institute of Malaria Research (ICMR-NIMR), New Delhi, India. We performed a cross-sectional analysis on the available clinical data of patients who came with fever at the clinic of ICMR-NIMR, New Delhi in September 2016. We used patients’ clinical records for labeling a case as probable dengue, probable chikungunya, or both. The clinic observed a standard protocol wherein patients with fever duration ≤ 5 days were tested for dengue NS1 antigen (Panbio Dengue Early ELISA kit, Standard Diagnostics Inc., Republic of S. Korea) and those with fever for > 5 days were tested for dengue- and chikungunya-specific IgM antibodies (MAC ELISA kit supplied by ICMR-National Institute of Virology, Pune, India). All tests were performed as per the manufacturers’ instructions and samples with > 11 Panbio units were interpreted as dengue NS1 positive. For IgM ELISA, sample optical density (OD) was taken at 450 nm and samples with OD ≥ three times the OD of negative control were considered as “positive”. Lab diagnosis was correlated with clinical features suggestive of chikungunya and dengue.

We used Stata 15 (StataCorpStata Statistical Software: Release 16. College Station, TX: StataCorp LLC) for data analyses wherein continuous variables were compared using analysis of variance if they followed normal distribution, else Kruskal–Wallis test was performed. All categorical variables were compared using the chi-square/Fisher exact test.

Independent variables included four non-clinical (age, gender, marital status, education, duration of fever) and 39 clinical parameters whereas dependent variables included the following four categories: only-dengue (NS1 antigen and/or dengue IgM positive but chikungunya IgM negative), only-chikungunya (NS1 antigen and dengue IgM negative but chikungunya IgM positive), both dengue and chikungunya (NS1 antigen and/or dengue IgM positive and chikungunya IgM positive), neither dengue nor chikungunya (NS1 antigen and dengue IgM negative and chikungunya IgM negative). We used bivariate analysis for estimating the association between variables followed by multivariate analysis on those variables that were found statistically significant in the bivariate analysis at 5% level of significance (*p* value < 0.05 was considered statistically significant). Direction and strength of the association were estimated by calculating the relative risk ratio (RRR) using logistic regression.

## Results

A total of 978 patients with fever or a history thereof were reported in the fever clinic of ICMR-NIMR, New Delhi in September 2016. The socio-demographic descriptive profile of patients is shown in Table 1 whereas the association of relevant socio-demographic variables and duration of fever with four dependent outcomes is shown in Table 2. Out of 978, ~ 56% patients were male and ~ 60% (588) were only educated up to higher secondary level with 17% never attended any formal schooling. Almost 50% of the patients had no formal employment. Among 978 patients, 541 (55%) patients had neither dengue nor chikungunya; 23 (2%) had only-dengue, 340 (35%) had only-chikungunya and 74 (8%) patients were found positive for both dengue and chikungunya. The mean age of the patients varied significantly between these four clinical outcomes with higher mean age for chikungunya infections either alone (31.1 years) or with dengue (29.4 years). The mean duration of fever also differed between these groups with lesser duration (5 days; 3–8 days’ range) in patients who neither had dengue nor chikungunya.

**Table 1:** Basic socio-demographic profile of patients

Variables	Classifications	Number of patients *n* (%)
Gender	Total	978 (100)
	Male	543 (55.5)
	Female	435 (44.5)
Marital status	Total	978 (100)
	Ever Married	567 (57.8)
	Unmarried	116 (11.9)
	Below the legal age for marriage	297 (30.4)
Educational status	Total	978 (100)
	Up to primary school	127 (13.0)
	Up to middle school	152(15.5)
	Up to higher secondary	309 (31.6)
	Graduate and above	181 (18.5)
	Not attended school	162 (16.6)
	Below the age of schooling	47 (4.8)
Occupation of the respondent	Total	931 (100)
	Unemployed	226 (23.1)
	Home maker	263 (26.9)
	Labourer	26 (2.7)
	Own work / business	297 (30.4)
	Below the age of occupation	166 (17.0)

**Table 2:** Bivariate analysis of socio-demographic variables and fever duration

Variables	Neither dengue nor chikungunya (*N = *541)	Only dengue (*N = *23)	Only chikungunya (*N = *340)	Both dengue and chikungunya (*N = *74)	*p* value
Age (years)	27.3 ± 15.9	24.0 ± 12.9	31.1 ± 15.7	29.4 ± 15.5	0.002
Gender, male	309 (57.1)	15 (65.2)	183 (53.8)	36 (48.6)	0.355
Education level					0.038
Not eligible	34 (6.3)	1 (4.3)	9 (2.6)	3 (4.0)	
Up to primary	79 (14.6)	3 (13.0)	38 (11.2)	7 (9.5)	
Up to middle	89 (16.4)	5 (21.7)	49 (14.4)	9 (12.2)	
Up to high school	157 (29.0)	11 (47.8)	111 (32.6)	30 (40.5)	
Duration of fever (days)	5 (3–8)	7 (5–10)	7 (6–12)	8 (6–10)	0.001

We noted 13 out of 39 independent clinical variables (33%) to be in statistically significant positive association (predictors) with only-chikungunya in bivariate analysis (Table 3). These included, in decreasing order of predictor RRRs, in brackets: bleeding gums (4.56), joint swelling (3.24), fever with joint pain in general (3.0), itching (2.93), rash (2.71), red spots (2.47), joint pain in hands (2.11), joint pain in elbows (1.96), loss of taste (1.72), red eyes (1.70), joint pain in ankles (1.62), joint pain in wrists (1.56), and weakness (1.35). Out of these, only joint pain in general (4.06), weakness (2.90), joint swelling (2.82), joint pain in elbows (2.34), rashes (2.25), and itching (1.76) and in addition pain in chest (3.40) were significantly associated with patients with both dengue and chikungunya. Only four clinical features (red eyes, photophobia, vomiting sensation and bleeding gums) were positively associated with only-dengue. Subsequently multivariate analyses (Table 4) on the 13 variables positively associated with chikungunya alone revealed that the most significant chikungunya predictors include: bleeding gums (RRR 6.23; 95% CI 1.29–30.07), joint swelling (RRR 1.95; 95% CI 1.35–2.80), itching (RRR 1.89; 95% CI 1.31–2.73), and joint pain in elbows (RRR 1.70; 95% CI 1.17–2.48) as were found statistically significantly associated with chikungunya only. Joint pain in elbows was also found significantly associated with both chikungunya and dengue (RRR 1.85; 95% CI 1.20 to 3.34). Weakness had significantly association with both dengue and chikungunya with RRR of 1.9 (95% CI 1.05–3.45). None of the 13 variables were found to be associated with dengue only. The positive predictive value (PPV) of all the four clinical parameters in detecting chikungunya over dengue was found to be > 90% with joint swelling having the highest PPV of 98.5%, followed by elbow pain (96.5%), joint pain (95%), itching (94.6%) and bleeding gums (91.7%).

**Table 3:**
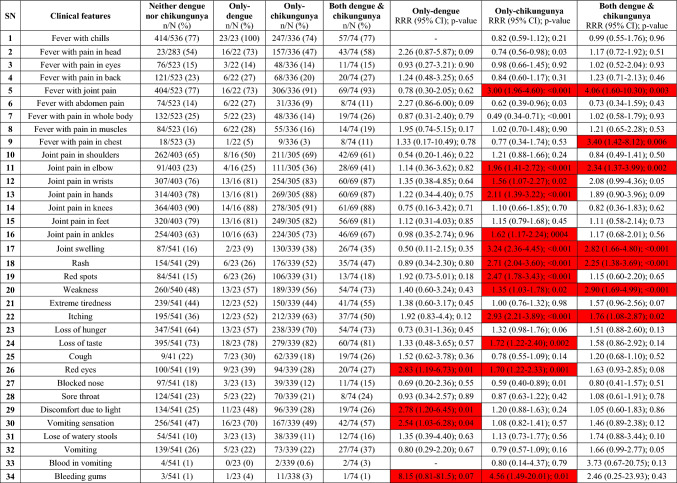
Bivariate analysis of clinical variables: Comparison of the 39 independent clinical variables which were reported by patients with only-dengue, only-chikungunya and both dengue and chikungunya

Patients with neither dengue nor chikungunya are used as controls. Relative risk ratio (RRR) with their 95% CI and *p* values are shown. Clinical features with statistically significant association (*p < *0.05) are highlighted red

**Table 4:**
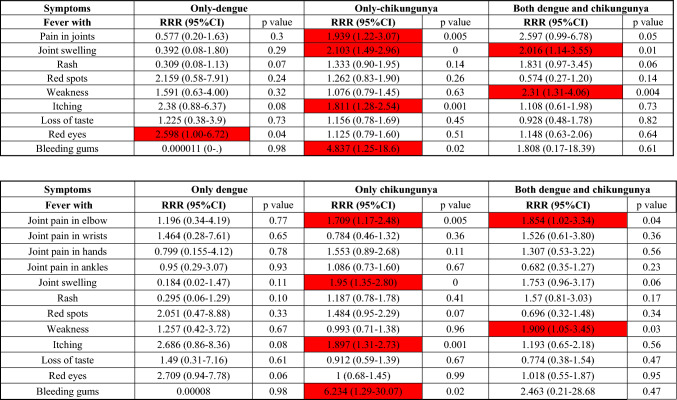
Multivariate analysis of clinical variables: Comparison of the independent clinical variables which were shown to be in statistically significant association with only chikungunya in bivariate analysis are shown

Relative risk ratio (RRR) with their 95% CI and *p* values are shown. Clinical features with statistically significant association (*p < *0.05) are highlighted red. The upper table has “pain in joints” as one variable whereas in the lower table, pain in joints is further categorized by the site of joint pain. The rest of the variables are same in both the tables

## Discussion

We found that a little less than half of the patients examined had chikungunya which justifies the need for a clinical predictor and we conclude that 4 clinical features (bleeding gums, joint swelling, itching, and joint pain in elbow) significantly increased the risk of having only CHIKV infection.

Joint involvement in chikungunya has been reported previously [32–42] but the sites were either not considered or differed between studies. Previous studies indicated that hands, wrist and ankles [43] or knees [44–47] were the most affected joints in chikungunya and could be one of its strongest predictors. This study reveals elbow pain (over other joints) as the most significant predictor of chikungunya. Although elbow pain was also significantly associated with patients having dengue and chikungunya both, it was not found associated with dengue only and therefore is highly plausible to be specific to chikungunya. The specificity and PPV of arthralgia has been reported to be ~ 99% and 85%, respectively [12] although dengue was not considered in this study. This study shows that the PPV of febrile arthralgia (irrespective of the type of joint involved) and pain in elbows (over other joints) correspond to 95% and 96.5%, respectively, when compared with dengue and therefore suggests their use as a strong predictor of chikungunya. However, the subjectivity of joint pains could be misleading as compared to joint swelling when it comes to selective prediction of chikungunya and, therefore, joint swelling could be a more valid clinical predictor than arthralgia [35] with a PPV of 98.5% as reported in this study.

Itching/pruritus (RRR 2.9; PPV 94.6%) also comes out to be a good clinical predictor of chikungunya. Studies have also reported pruritus to be significantly associated with chikungunya [12] but other AVDs including dengue were not compared. An Indian study reported itching in 50% of chikungunya cases but its use as clinical predictor of chikungunya in syndemics has not been evaluated [48]. Only one study could be found that concluded that pruritic skin was a clinical predictor of chikungunya against other febrile illnesses and dengue [40]. Bleeding gums (RRR 6.23; *p < *0.05) emerged out to be the strongest predictor (highest RRR) of chikungunya in this study. Not many studies have examined this but those that did, they focused on examination of oral manifestations of chikungunya. Oral lesions common in chikungunya with gingival bleeding amongst the top few manifestations [38, 49, 50]. However, bleeding manifestations are more often associated with dengue [34] that are less common in chikungunya [51] and despite significant association with chikungunya, it would not be clinically apt to use it as its predictor in a dengue–chikungunya syndemic setting. Further, the analyses have a limitation: bleeding gums was more strongly associated with dengue-only as compared to chikungunya-only group (RRR 8.15 versus 4.56; Table 2) along with photophobia and vomiting sensation that were found associated with dengue-only group and not with chikungunya. Multivariate analyses were done only on 13 variables that were associated with chikungunya only and hence did not consider photophobia and vomiting sensation. In addition, only 3% of chikungunya cases (11/338) had bleeding gums against 4% in dengue (1/23) and due to lesser number of events, the association might have been over-estimated.

Other symptoms including fever [45, 46, 52], polyarthralgia [12, 52], joint swelling [47] were also reported among CHIKV infected population in past studies. The presence of myalgia [12, 47, 53] was also observed among CHIKV-infected population but recent clinical records were unable to find any significant association with chikungunya.

## Conclusion

Segregating and prioritizing cases for chikungunya-specific lab diagnosis might be effective in dengue syndemic resource-limited settings. Although limited by the number of patients with dengue only, the findings from this study do conclude that joint involvement (joint pain in general, pain in elbows and joint swelling) can strongly predict, either singly or together, chikungunya for confirmatory diagnosis. Itching and bleeding gums can also predict chikungunya but they deserve further evaluation.

## Data Availability

The data may be made available on a reasonable request.

## Abbreviations

AVDs: Arboviral diseases
CHIKV: Chikungunya virus
CI: Confidence interval
DENV: Dengue virus
ELISA: Enzyme-linked immunosorbent assays
IFA: Indirect immunofluorescence assays
NS1 antigen: Non-structural 1 antigen
OD: Optical density
OPD: Out-patient department
PPV: Positive predictive value
RRR: Relative risk ratio
RT-PCR: Reverse transcriptase PCR
